# A Rare Coexistence of Klippel–Trenaunay Syndrome and Cardiac Sarcoidosis

**DOI:** 10.3390/biomedicines13061326

**Published:** 2025-05-29

**Authors:** Shriya Sharma, Aarti Desai, Hans Mautong, Patricia Mergo, Juan Leoni, Jose Ruiz, Rohan Goswami

**Affiliations:** 1 Division of Heart Failure and Transplant, Mayo Clinic, Jacksonville, FL 32224, USA; shriyasharmap@gmail.com (S.S.); desai.aarti@mayo.edu (A.D.); leoni.juan@mayo.edu (J.L.); ruizmorales.jose@mayo.edu (J.R.); 2 School of Health, Universidad Espíritu Santo-Ecuador, Samborondón 092301, Guayas, Ecuador; hansmautong@uees.edu.ec; 3 Division of Radiology, Mayo Clinic, Jacksonville, FL 32224, USA; mergo.patricia@mayo.edu

**Keywords:** Klippel–Trenaunay syndrome, cardiac sarcoidosis, heart failure, heart transplant

## Abstract

Klippel–Trenaunay (KT) syndrome and Cardiac Sarcoidosis (CS) are two distinct medical conditions that rarely coexist, each presenting unique challenges in diagnosis and management. Here, we present a rare case of a 30-year-old male with a history of KT syndrome complicated by recurrent deep vein thrombosis, who presented with symptoms of acute heart failure including shortness of breath, fatigue, dizziness, palpitations, and chest pain and was subsequently diagnosed with isolated CS. We discuss the importance of thorough clinical evaluation and multimodal diagnostic approaches in this complex scenario with overlapping symptoms and diagnostic dilemmas.

## 1. Background

Klippel–Trenaunay (KT) syndrome is characterized by a complex array of abnormalities, including cutaneous capillary malformations often found on an affected limb, abnormal development of deep and superficial veins, and limb asymmetry typically involving enlargement. It may also involve mixed vascular malformations encompassing capillary, venous, arterial, and lymphatic systems [1]. The estimated incidence is between two and five cases per 100,000, with a higher prevalence in males than females [2].

Sarcoidosis is a systemic inflammatory disease characterized by noncaseating granulomas, primarily affecting the lungs but also involving the heart, leading from silent myocardial involvement to symptomatic arrhythmia and heart failure. Despite improvements in prognosis, Cardiac Sarcoidosis (CS) remains potentially fatal, primarily due to arrhythmic events rather than heart failure [3].

To our knowledge, this is the first reported case of the coexistence of KT syndrome and CS. It presents diagnostic and therapeutic challenges due to overlapping clinical features and complications. We aim to highlight the complexities associated with managing these two distinct medical conditions in the first ever reported case of concurrent occurrence.

## 2. Case Presentation

A 30-year-old male, with a significant medical history including Klippel–Trenaunay syndrome affecting the left lower extremity, recurrent deep vein thrombosis (DVT) treated with enoxaparin 120 mg subcutaneous injection BD, and a dual-chamber pacemaker implanted a month prior for third-degree heart block. An echocardiogram performed at the time of pacemaker implantation was normal. He presented to our facility with symptoms of heart failure (HF) including worsening shortness of breath, fatigue, dizziness, palpitations, and chest pain.

### 2.1. Initial Workup

**Figure 1:**
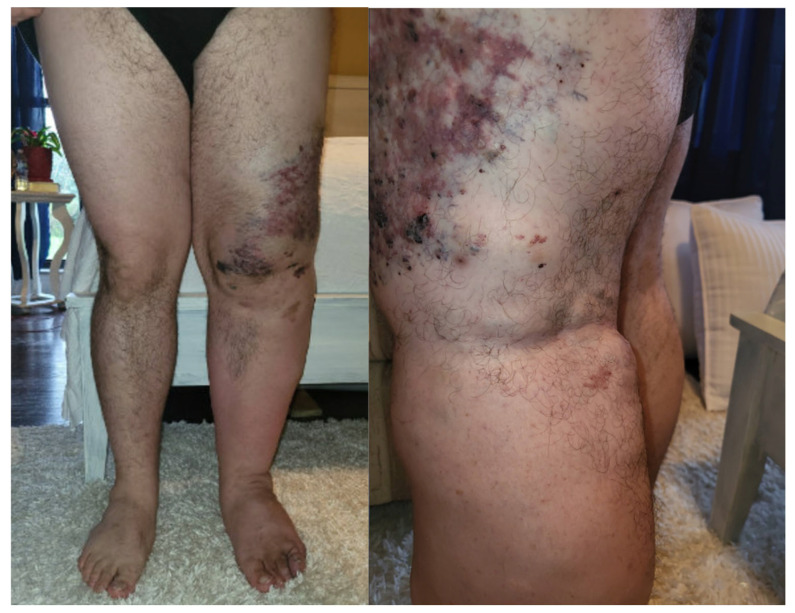
Hemihypertrophy of the left lower limb with bluish-purple discoloration of skin, port wine stain, and varicose veins.

Upon arrival, the patient’s vital signs were as follows: blood pressure of 124/92 mmHg, heart rate of 119 beats per minute, oxygen saturation of 96% on room air with a body mass index (BMI) of 38.64 kg/m^2^. Significant edema was noted in the left lower extremity compared to the right, consistent with known DVT and vascular malformations—port wine stain, varicosities (Figure 1).

**Figure 2:**
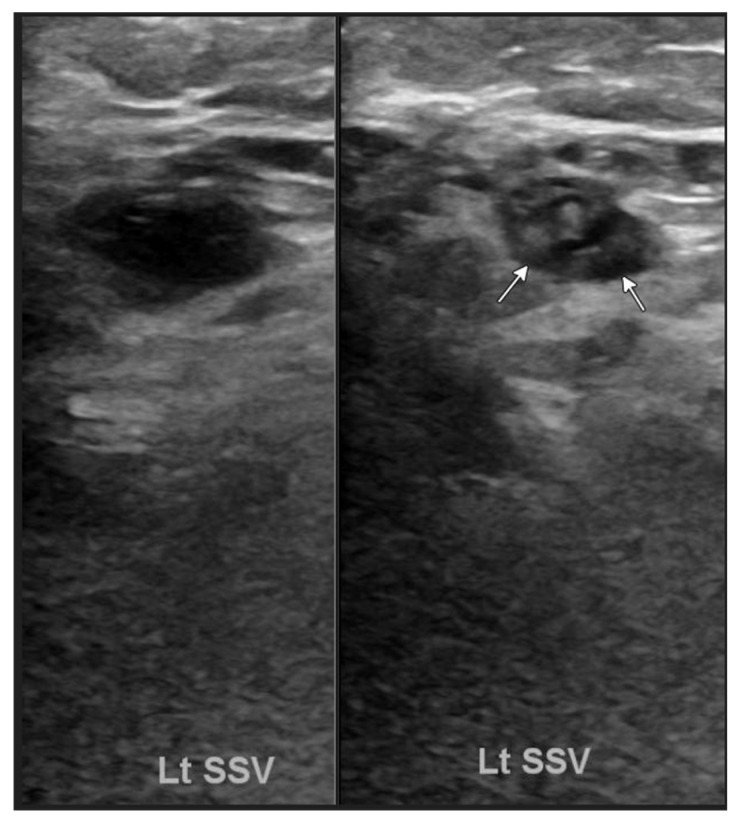
Ultrasound of the left lower extremity with and without compression applied demonstrates a nonocclusive superficial venous thrombus in the left small saphenous vein (arrows).

Initial laboratory results showed lactate levels of 2.6 mmol/L (reference range: 0.5–2.2 mmol/L), Troponin-T of 35 ng/L (reference range: ≤15 ng/L), B-type natriuretic peptide (BNP) of 1852 pg/mL, prothrombin time (PT) of 13.2 s (reference range: 9–13 s), and international normalized ratio (INR) of 1.2 (reference range: 0.8–1.2). An ultrasound of the left lower extremity, with and without compression applied, revealed a nonocclusive superficial venous thrombus in the left small saphenous vein (Figure 2). Chest X-ray revealed a stable enlarged cardiac/pericardial silhouette and stable mediastinal contours. An electrocardiogram (EKG) revealed sinus tachycardia with ventricular pacing and premature ventricular contractions. The echocardiogram revealed a significantly enlarged left ventricular chamber size with the presence of regional wall motion abnormalities (basal septal akinesis/dyskinesis) with a left ventricular ejection fraction of 28%. Additionally, left ventricular internal diameter was 59 mm in diastole, 51 mm in systole, and severe mitral valve regurgitation was identified. Basal septal akinesis/dyskinesis further raised concerns for possible CS. Right heart catheterization demonstrated elevated pressures, with a right atrial pressure of 9 mmHg, right ventricular pressure of 42/12 mmHg, pulmonary artery pressure of 38/27 mmHg (31 mmHg), and pulmonary capillary wedge pressure of 24 mmHg, indicative of acute heart failure.

**Figure 3:**
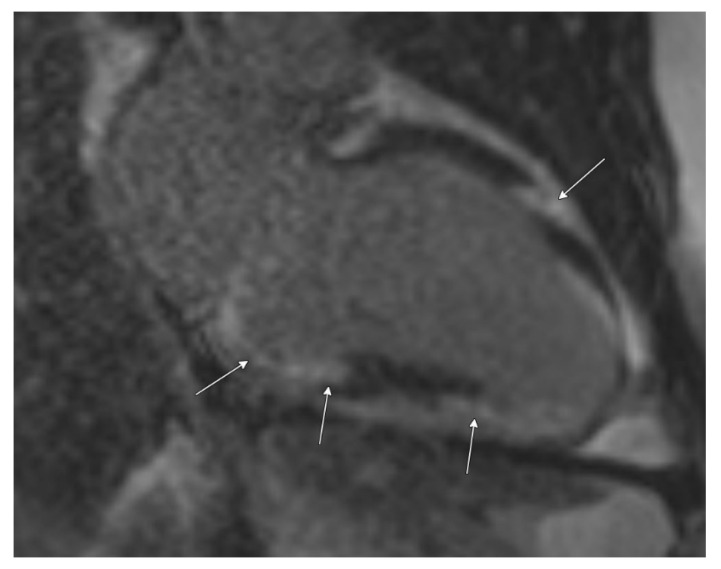
Cardiac MRI with late post-gadolinium-enhanced inversion recovery imaging in a two-chamber view demonstrates extensive and multifocal left ventricular myocardial late enhancement in a mid-wall and epicardial predominant distribution, particularly involving the basal inferior wall and the mid-anterior wall (arrows). Abnormal late enhancement at the apex is more diffuse in distribution.

**Figure 4:**
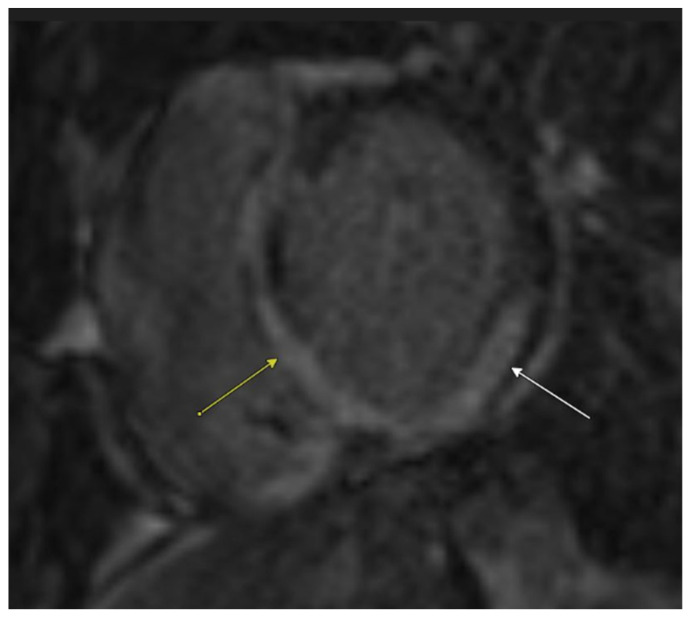
Cardiac MRI with late post-gadolinium-enhanced inversion recovery imaging in a short axis view at the base demonstrates extensive abnormal left ventricular myocardial late enhancement in a mid-wall and epicardial predominant distribution, particularly involving the basal inferior wall, septum (yellow arrow), and inferolateral wall (white arrow).

**Figure 5:**
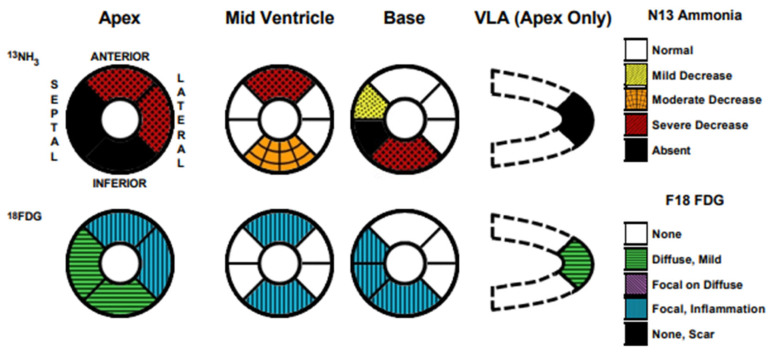
Positron Emission Tomography (PET) scan demonstrating a prominent diffuse myocardial FDG uptake with concomitant perfusion abnormalities, illustrated in the bull’s eye diagram.

Cardiac MRI confirmed biventricular dysfunction, with left ventricular ejection fraction of 12%, right ventricular ejection fraction of 18%, basal septal akinesis/dyskinesis, and extensive abnormal late gadolinium enhancement, further supporting the suspicion of CS (Figure 3 and Figure 4). While there were no clinical signs of non-sarcoid inflammatory cardiomyopathy, a Positron Emission Tomography (PET) scan showed a very large area of diffuse myocardial FDG uptake with associated perfusion abnormalities, as described in the bull’s eye diagram, further supporting the diagnosis of CS (Figure 5). Except for cardiac abnormalities, a full-body CT PET was negative. CT coronary angiography revealed patent coronary arteries, while CT pulmonary angiography showed chronic nonocclusive segmental acute pulmonary embolus in the posterior right lower lobe without right heart strain. Ultrasonography Doppler of the lower extremities identified subacute nonocclusive superficial venous thrombosis involving the small saphenous vein, consistent with the patient’s history of KT syndrome.

This combination of findings is compatible with CS with active inflammation and associated edema, fibrosis, and/or scar in the appropriate clinical context.

### 2.2. Diagnosis and Management

The patient was diagnosed with new-onset acute heart failure (AHF) and started on diuretic therapy with intravenous furosemide 40 mg twice daily and guideline-directed medical therapies (GDMTs) including Empagliflozin 10 mg daily, Metoprolol 25 mg daily, sacubitril/valsartan 24–26 mg twice daily, and Spironolactone 25 mg daily. Steroid therapy for sarcoidosis was not pursued due to concern for the development of acute kidney injury.

For chronic venous malformations and recurrent DVT in the left lower extremity, the patient was initially treated with Apixaban but switched to Enoxaparin due to recurrent DVT. The multidisciplinary team recommendations included heparin infusion and subsequent transition to dabigatran 150 mg twice daily with close renal function monitoring, as well as compression therapy at 30–40 mm Hg for pain management of the left leg and outpatient follow-up.

Dual-chamber pacemaker device interrogation revealed normal function with sinus tachycardia and second-degree atrioventricular block type II.

### 2.3. Follow-Up

Follow-up with vascular surgery for venous malformations and implementation of weight loss strategies were recommended as outpatient specialty measures, given that a BMI below 35 kg/m^2^ is a prerequisite for heart transplant candidacy. The patient remained on GDMT for six months to achieve stabilization of heart failure symptoms and successfully underwent orthotopic heart transplantation without any complications. Currently, 4 months post-transplant, patient is stable without signs of graft rejection.

## 3. Discussion

We present a case of KT syndrome complicated by recurrent DVT and isolated CS, with progression to AHF. This case illustrates the diagnostic and therapeutic challenges associated with the coexistence of KT syndrome and CS. The complex interplay between vascular abnormalities in KT syndrome and cardiac involvement in sarcoidosis highlights the need for a multidisciplinary approach.

### 3.1. Diagnosis and Management of KT Syndrome

KT syndrome is a rare congenital disorder characterized by a triad of cutaneous capillary malformations, bone and soft tissue hypertrophy, and venous and lymphatic malformations [4]. Our patient presented the classic manifestation of Klippel–Trenaunay syndrome with port wine stain, prominent varicose veins, and soft tissue hypertrophy. These physical findings, along with a history of recurrent DVT involving the affected limb, were consistent with a diagnosis of KT syndrome.

The vascular abnormalities associated with KT syndrome predispose patients to thromboembolic events, such as DVT. Venous stasis in the lower limb increases the risk of thrombus formation and subsequent pulmonary thromboembolism (PTE). Due to the persistent thrombus formation, recurrent thromboembolic events are prevalent in these patients along with the need for lifelong anticoagulation therapy following the initial occurrence of DVT or PTE. In our patient, despite appropriate anticoagulation with apixaban, recurrent DVT occurred, in addition to having a low output state with heart failure—potentiating the risk of thrombus formation. This interplay highlights the challenging nature of managing thrombotic complications in KT syndrome. Furthermore, the potential development of chronic thromboembolic pulmonary hypertension (CTEPH), a rare complication following PTE, should be considered as a potential cause of pulmonary hypertension and worsening heart failure in these patients. Studies have reported a cumulative incidence of CTEPH ranging from 0.1% to 9.1% within the initial two years after experiencing symptomatic PTE. Despite anticoagulation therapy, recurrent thromboembolic events remain common in Klippel–Trenaunay syndrome patients. Differential diagnosis of CTEPH from other causes of pulmonary hypertension can be achieved through ventilation–perfusion lung scanning and widely utilized chest CT scans for diagnostic purposes [5]. Treatment of KT syndrome is conservative unless complications occur.

### 3.2. Diagnosis and Management of CS

Concurrently, our patient had sarcoidosis, a systemic inflammatory disease of unknown etiology, characterized by noncaseating granulomas affecting various organs including the lung, heart, liver, spleen, skin, and eyes. In systemic sarcoidosis, the lungs are the most affected organs, with an incidence exceeding 90%, while cardiac involvement is observed in only 2–3% of patients [3]. In contrast, CS presents with symptoms in only 5% of sarcoidosis patients, yet cardiac involvement is identified in 25% to 28% of cases, a proportion that is rising with the use of advanced cardiovascular imaging methods [6]. Despite lacking typical systemic manifestations of sarcoidosis, such as pulmonary involvement or lymphadenopathy, the patient exhibited significant cardiac manifestations, including conduction abnormalities, ventricular tachycardia, and severe biventricular dysfunction. Our patient was implanted with a dual-chamber pacemaker at a young age of 30, 2 months prior to the diagnosis of CS. Echocardiography performed at the time of pacemaker implant did not reveal suspecting features of CS. However, cardiac MRI performed 2 months post-pacemaker implantation confirmed diagnosis.

Typical echocardiographic findings in CS include multiple regional wall motion abnormalities (RWMAs) that do not correspond to specific coronary artery territories. Thinning of the basal anterior septum is particularly indicative of CS. Cardiac MRI aids in distinguishing between ischemic and non-ischemic cardiomyopathies based on the pattern of late enhancement. While various lesions can be classified as non-ischemic cardiomyopathy, a patchy and multiple DE pattern strongly suggests CS. Additionally, late enhancement may be observed in segments without RWMAs, further supporting a diagnosis of CS [7]. The patient’s echocardiogram revealed multiple RWMAs, and cardiac MRI showed extensive late gadolinium enhancement, consistent with CS and indicating ongoing myocardial injury. The patchy nature of CS poses challenges in obtaining definitive histological confirmation, with endomyocardial biopsy yielding a diagnosis in approximately 20% of cases [3].

The currently accepted diagnostic criteria include the World Association for Sarcoidosis and Other Granulomatous Disorders (WASOG) 2014 criteria, the Heart Rhythm Society (HRS) 2014, and the Japanese Circulation Society (JCS) 2016 criteria. As per the WASOG criteria of diagnostic probability, our patient satisfied several diagnostic criteria outlined as ‘at-least probable’ including high-degree AV block and imaging abnormalities, features that overlap with the diagnostic criteria outlined by the HRS. Multiple major clinical criteria from the 2016 JCS guidelines are also met, including high-grade FDG uptake on cardiac PET scan consistent with active myocardial inflammation, abnormal late gadolinium enhancement (LGE) on cardiac MRI, and the absence of other systemic sarcoidosis or identifiable causes of granulomatous myocarditis (e.g., tuberculosis, fungal or bacterial infections, autoimmune conditions) [7,8,9].

The management of CS involves a multifaceted approach, incorporating immunosuppressive therapy and GDMT for heart failure and arrhythmia. Nonspecific immunosuppression using corticosteroids is the primary treatment approach for CS. Corticosteroids act by suppressing the production of cytokines involved in granuloma formation, such as TNF-α and IFN-γ. The treatment goal is to reduce myocardial inflammation, thereby limiting the development of myocardial fibrosis and associated complications like re-entrant ventricular arrhythmias, heart block, and worsening of left ventricular dysfunction and heart failure [10,11].

The JCS recommends initiating treatment with an initial prednisolone dose of 30 mg daily or 60 mg on alternate days for 4 weeks, followed by a gradual tapering regimen of 5 mg monthly to achieve a maintenance dose of 5–10 mg daily or 10–20 mg on alternate days by 6 months [7]. However, it is important to note that steroid or immunosuppressant therapy may be reserved for patients with relatively preserved LV function (EF > 35%), as these therapies are aimed at preserving rather than recovering deteriorated LV function [10]. They may also be ineffective in preventing malignant ventricular arrhythmias in patients with severely depressed LV function, as observed in our case.

In addition to immunosuppression, patients with CS should receive GDMT for managing electrophysiological and heart failure manifestations. In cases of reduced LV systolic function, standard heart failure GDMT typically includes beta-blockers, renin–angiotensin system blockade with angiotensin-converting enzyme inhibitors, angiotensin receptor blockers, or a neprilysin inhibitor–angiotensin receptor combination (sacubitril/valsartan). Symptomatic patients may benefit from adding a mineralocorticoid receptor antagonist, while diuretics are used to manage volume overload symptoms [12].

Device therapy with permanent pacemakers and/or an implantable cardioverter defibrillator (ICD) is an essential component of the therapeutic approach to patients with CS and arrhythmic events. The implantation of a permanent pacemaker is the definitive treatment for AV nodal disease in CS and may be appropriate even in cases of transiently recovered AV conduction. Furthermore, due to the risk of sudden cardiac death in CS, consideration should be given to implanting an ICD when permanent pacing is indicated. This has further been shown in work by Nordenswan et al., which consisted of 325 CS patients, 143 of whom had high-grade AV block—also benefiting from ICD therapies [13]. ICDs have both pacemaker and defibrillator functions, and the 2017 American Heart Association/American College of Cardiology and HRS guideline recommends ICD implantation in patients with evidence of extensive myocardial scarring on cardiac MRI or PET scan, even in cases where the ejection fraction is >35% [13,14].

### 3.3. Complexities Associated with the Coexistence of KTS and CS

To our knowledge, this is the first reported case of KT syndrome and CS. The simultaneous occurrence of these two conditions poses significant diagnostic and management challenges. The chronic inflammation and systemic vascular abnormalities in KT syndrome may predispose patients to a pro-inflammatory state [15], potentially exacerbating the granulomatous process of sarcoidosis. Alternatively, the high incidence of thromboembolic events found in KT syndrome (17–22%) can potentially result in chronic thromboembolic pulmonary hypertension (CTEPH) [16,17], which would certainly worsen heart failure manifestations in patients with concomitant CS.

This case underscores the importance of maintaining a high index of suspicion for concurrent systemic conditions in patients with complex presentations. Advanced imaging modalities, such as cardiac MRI and FDG-PET, combined with histological confirmation (when available), are pivotal for diagnosing CS in this context. Multidisciplinary management, including cardiologists, rheumatologists, and vascular specialists, is essential to optimize outcomes. Further research is warranted to explore potential pathophysiological links and refine treatment strategies for such rare coexisting conditions.

## 4. Conclusions

Our case highlights the complexity of managing Klippel–Trenaunay syndrome and isolated Cardiac Sarcoidosis together in the setting of acute heart failure. Most patients with KT syndrome should be managed conservatively for prevention and treatment of complications. It is important to consider CS in patients presenting with conduction abnormalities, especially in the absence of typical clinical manifestations of CS. The interplay between the vascular abnormalities of KT syndrome and the granulomatous inflammation of sarcoidosis necessitates advanced imaging modalities, coupled with a high index of clinical suspicion and a multidisciplinary approach to ensure timely diagnosis and appropriate management of this complex condition.

## Data Availability

The raw data supporting the conclusions of this article will be made available by the authors on request.

## Abbreviations

CS	Cardiac Sarcoidosis
CTEPH	Chronic thromboembolic pulmonary hypertension
DVT	Deep vein thrombosis
GDMT	Guideline-directed medical therapy
ICD	Implantable cardioverter defibrillator
KT Syndrome	Klippel–Trenaunay syndrome
PET	Positron Emission Tomography
PTE	Pulmonary thromboembolism
RWMA	Regional wall motion abnormalities
